# The efficacy of thymosin alpha 1 for severe sepsis (ETASS): a multicenter, single-blind, randomized and controlled trial

**DOI:** 10.1186/cc11932

**Published:** 2013-01-17

**Authors:** Jianfeng Wu, Lixin Zhou, Jiyun Liu, Gang Ma, Qiuye Kou, Zhijie He, Juan Chen, Bin Ou-Yang, Minying Chen, Yinan Li, Xiaoqin Wu, Baochun Gu, Lei Chen, Zijun Zou, Xinhua Qiang, Yuanyuan Chen, Aihua Lin, Guanrong Zhang, Xiangdong Guan

**Affiliations:** 1 Department of Critical Care Medicine, The First Affiliated Hospital of Sun Yat-sen University, 58 Zhongshan Er Road, Guangzhou 510080, Guangdong Province, PR China; 2 Department of Critical Care Medicine, Foshan First Municipal People's Hospital, 81 Lingnan North Road, Foshan 528000, Guangdong Province, PR China; 3 Department of Critical Care Medicine, Guangzhou First Municipal People's Hospital, 1 PanFu Road, Guangzhou 510180, Guangdong Province, PR China; 4 Department of Critical Care Medicine, Sun Yat-sen University Cancer Center, 651 Dongfeng Road East, Guangzhou 510060, Guangdong Province, PR China; 5 Department of Critical Care Medicine, The Sixth Affiliated Hospital of Sun Yat-sen University, 26 Yuancun Erheng Rd, Guangzhou 510655, Guangdong Province, PR China; 6 Department of Critical Care Medicine, The Second Affiliated Hospital of Sun Yat-sen University, 107 Yan-Jiang West Road, Guangzhou 510120, Guangdong Province, PR China; 7 School of Public Health, Sun Yat-sen University, 74 Zhongshan Er Road, Guangzhou 510080, Guangdong Province, PR China

## Abstract

**Introduction:**

Severe sepsis is associated with a high mortality rate despite implementation of guideline recommendations. Adjunctive treatment may be efficient and require further investigation. In light of the crucial role of immunologic derangement in severe sepsis, thymosin alpha 1 (Tα1) is considered as a promising beneficial immunomodulatory drug. The trial is to evaluate whether Tα1 improves 28-day all-cause mortality rates and immunofunction in patients with severe sepsis.

**Methods:**

We performed a multicenter randomized controlled trial in six tertiary, teaching hospitals in China between May 12, 2008 and Dec 22, 2010. Eligible patients admitted in ICU with severe sepsis were randomly allocated by a central randomization center to the control group or Tα1 group (1:1 ratio). The primary outcome was death from any cause and was assessed 28 days after enrollment. Secondary outcomes included dynamic changes of Sequential Organ Failure Assessment (SOFA) and monocyte human leukocyte antigen-DR (mHLA-DR) on day 0, 3, 7 in both groups. All analyses were done on an intention-to-treat basis.

**Results:**

A total of 361 patients were allocated to either the control group (*n *= 180) or Tα1 (*n *= 181) group. The mortalities from any cause within 28 days in the Tα1 group and control group were 26.0% and 35.0% respectively with a marginal *P *value (nonstratified analysis, *P *= 0.062; log rank, *P *= 0.049); the relative risk of death in the Tα1 group as compared to the control group was 0.74 (95% CI 0.54 to 1.02). Greater improvement of mHLA-DR was observed in the Tα1 group on day 3 (mean difference in mHLA-DR changes between the two groups was 3.9%, 95% CI 0.2 to 7.6%, *P *= 0.037) and day 7 (mean difference in mHLA-DR changes between the two groups was 5.8%, 95% CI 1.0 to 10.5%, *P *= 0.017) than in the control group. No serious drug-related adverse event was recorded.

**Conclusions:**

The use of Tα1 therapy in combination with conventional medical therapies may be effective in improving clinical outcomes in a targeted population of severe sepsis.

**Trial registration:**

ClinicalTrials.gov NCT00711620.

## Introduction

Severe sepsis is an important cause of admission to intensive care units (ICUs) throughout the world and is characterized by high mortality in adults [1-3]. Severe sepsis is diagnosed in more than 750,000 people annually in the United States, of whom 215,000 will die [3]. Reported mortality rates of severe sepsis ranged from 28% to 35.5% [3-7]. In spite of the adoption of therapeutic bundles based on Surviving Sepsis Campaign (SSC) guidelines, mortality is reported to be about 30% [4]. The key role of immunologic derangement in the course and the poor outcome has led to an increased interest in immunotherapy [8,9]. Thymosin alpha 1 (Tα1) is a naturally occurring thymic peptide first described and characterized by Goldstein *et al. *[10]. It acts as an endogenous regulator of both the innate and adaptive immune systems [11]. It is used worldwide for treating diseases associated with immune dysfunction including viral infections such as hepatitis B and C, certain cancers, and for vaccine enhancement [12,13]. Notably, recent development in immunomodulatory research has indicated the beneficial effect of Tα1 treatment in septic patients. However, the results of these studies should be viewed with caution due to their small sample sizes and use of more than one drug as therapeutic intervention [14-16]. This multicenter randomized controlled trial was implemented to determine the efficacy of Tα1 in treating severe sepsis.

## Material and methods

We did a prospective, controlled, single-blinded, multicenter randomized clinical trial, which was conducted in the ICUs of six tertiary, teaching hospitals. The ethics committee of the First Affiliated Hospital of Sun Yat-sen University approved the protocol (200815). Written informed consents were obtained from the patients or next of kin for patients unable to consent. The trial was registered with ClinicalTrials.gov, number NCT00711620.

### Patients

From May 12, 2008 to Dec 22, 2010 patients diagnosed with severe sepsis admitted to ICUs were enrolled in the trial. The criteria for severe sepsis were a modification of those defined by Bernard *et al. *(see Additional file 1) [7]. Patients were eligible for study inclusion if they had a known or suspected infection based on clinical data at the time of screening and if they had two or more signs of systemic inflammation and sepsis-induced dysfunction of at least one organ or system. Exclusion criteria are summarized in Additional file 2.

### Randomization and masking

To reduce the impact on the results from heterogeneity of severe sepsis and inter-hospital variation in patient sources as much as possible, stratification by investigative center in combination with computer-generated block randomization (block size = 8) according to the sequence of recruitment was employed in the enrollment process. The method of randomization and block size were blinded until the data analysis was finished completely. Clinicians who enrolled the subjects were not involved in data collection. Eligible patients were randomly assigned in a 1:1 ratio in each hospital with four in each block assigned to receive the study drug and the other four to the control group after telephone verification through a randomization center. The allocation sequence was concealed from the researchers. To prevent advance knowledge of treatment assignment and subversion of the allocation sequence, trial entry sheet of the case report form (CRF) was filled out and informed consent was obtained before disclosing the unique participant number and the allocated group; the unique number generated could not be changed and deleted afterward. We used normal saline as placebo. Patients were blinded to the treatment assignments. All statistical analysis was done with masking maintained.

### Study drug administration and sepsis management

In the Tα1 group, patients received subcutaneous injections of 1.6 mg Tα1 (ZADAXIN™, SciClone Pharmaceuticals, Foster City, CA, USA) twice per day for five consecutive days, then once per day for two consecutive days. Prior to administration, the lyophilized powder is to be reconstituted with 1 ml of the provided diluent (sterile water for injection). After reconstitution, the final concentration of Tα1 is 1.6 mg/ml. In the control group, patients received subcutaneous injections of 1 mL normal saline twice per day for five consecutive days, then once per day for two consecutive days. According to trial protocol, therapy had to be started within 4 hrs after enrollment.

The treating physicians dictated patient care to current international guidelines [17], including adequate empiric antibiotic therapy based on current recommendations, ventilation regimen (pressure control mode), blood glucose control, resuscitation and hemodynamic support, organ support, sedation or analgesia as needed and adequate nutrition. Empirical antibiotic therapy was considered adequate when at least one effective drug was included in the empirical antibiotic treatment within the first 24 hrs of the admission to the ICU and the optimal dose and the correct route of administration were in accordance with medical standards and in ICU survivors without microbiologically detected microorganism in bloodstream or focus. When the empirical antibiotic therapy had to be changed after microbiological detection of microorganism, it was considered inadequate, whereas in non-survivors without microbiologically detected microorganism in bloodstream or focus it was considered not evaluable [18-20].

### Outcomes and data collection

The primary efficacy end point was death from any cause and was assessed 28 days after the initiation of treatment assignment. Secondary outcomes included dynamic changes of Sequential Organ Failure Assessment (SOFA), CD^4+^/CD^8+ ^and monocyte human leukocyte antigen-DR (mHLA-DR) expression measured on day 0 (the day of enrollment), 3 and 7 in both groups. All mHLA-DR measurements were done in the center laboratory of the First Affiliated Hospital of Sun Yat-sen University. 1 ml unprocessed EDTA whole blood was stored on ice at once after drawing and was transferred to the center laboratory as soon as possible to guarantee measurement within 3 hrs after blood drawing. The method of measuring mHLA-DR was mentioned in our previous paper [21]. Once patients were enrolled, data including demographic characteristics, microbiological findings (primary infection source and the identified microorganisms) and comorbidities were collected when available. The following clinical parameters were recorded on specific days after enrollment: on day 0, the severity as assessed by the Acute Physiology and Chronic Health Evaluation II (APACHE II); on day 0, 3, 7, SOFA, hematologic and biochemical findings, results of mHLA-DR, CD^4+^/CD^8+ ^tests. The time of the first organ dysfunction was retrospectively estimated according to objective data such as blood gas analysis when the patient was enrolled.

### Statistical analysis and sample size

Based on a previous study [22], a sample size of 334 patients was required to show a reduction in 28-day mortality rate from 50% to 35% by Tα1 treatment, with a two-sided test (α error = 5%; power = 80%). Considering a possible drop-out rate of 10%, the trial would need to enroll 368 patients in total. Demographic data, outcome data and other laboratory parameters were summarized by frequency for categorical variables and mean ± standard deviation (SD) or median with interquartile range (IQR) for continuous variables. Proportions were compared with chi-square test or Fisher's exact test. Continuous variables were tested by means of *t *test with normal distribution or Wilcoxon rank-sum test with non-normal distribution. The comparison of primary outcome between two groups was performed by means of Cochran-Mantel-Haenszel test, in which patients were stratified on a number of baseline covariates such as mHLA-DR, scores of APACHE and SOFA, surgical and cancer history, sex and age. The corresponding relative risks (RRs) with 95% confidence intervals (CIs) were computed with logit-adjusted method. Kaplan-Meier estimates without adjustment for baseline covariates were used for survival time analysis, and log-rank tests for comparison. To estimate mean changes from baseline in laboratory parameters, linear mixed models for repeated measures were employed, taking into account the clustering of participating centers and repeated measurements within patients. This model included terms for baseline measurement, treatment group, visit, and treatment × visit interaction. Least-squares means with 95% CIs were reported. We also analyzed the efficacy parameters of the study drug in different prespecified subgroups. The heterogeneity of treatment effects among subgroups was assessed with use of interaction tests. Consistent with the intention-to-treat principle, all analyses were based on all available population, consisting of those with a baseline and at least one post-baseline efficacy measurement, neither making any assumption nor imputing the missing data. All statistical analyses were done with the SAS software (SAS 9.1.3; SAS Institute Inc., Cary, NC, USA). Two-sided *P *values were reported and a *P *value less than 0.05 was considered as statistically significant.

## Results

### Study profile

**Figure 1:**
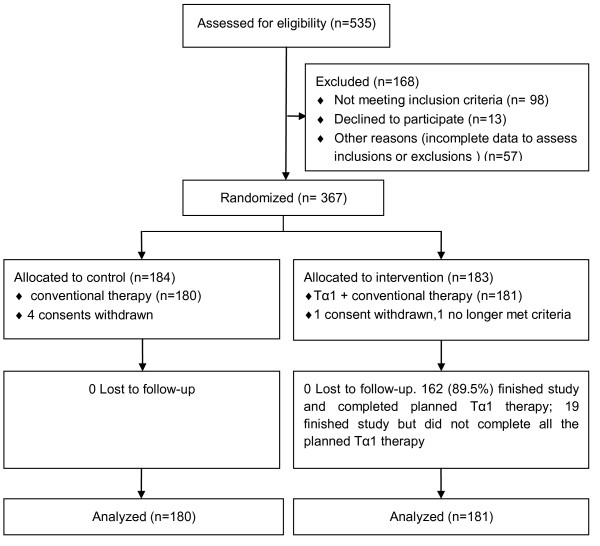
**Study profile**. Tα1, thymosin alpha 1.

Between May 12, 2008 and Dec 22, 2010, 367 eligible patients were randomized (Figure 1). In the Tα1 group, two patients were excluded: one patient withdrew the consent after being diagnosed with typhus and was transferred to the infectious disease hospital immediately; in the other case, consent was withdrawn before the infusion. In the control group, consents were withdrawn after the enrollment in four cases. A total of 361 randomized patients were followed up for the entire 28-day study period without drop-out. Of 181 patients in Tα1 group, 162 patients completed the trial in adherence with the protocol regarding the use of drugs, while the other 19 patients received at least 1.6 mg Tα1 but their treatments did not fully adhere to the protocol because they were transferred out of ICU.

### Baseline data

Both groups had similar characteristics in most demographic and baseline variables (Table 1), although patients in the Tα1 group had a longer period between the time of first organ dysfunction observed and the time of enrollment (42 hrs vs. 28 hrs, *P *= 0.003). Nearly 80% of the patients had at least two dysfunctional organs at the time of enrollment. The pulmonary and cardiovascular systems were the most commonly affected organ systems with an incidence of 94.7% and 65.7% respectively. The most common sites of infection were lung and abdomen, with an incidence of 74.5 and 27.4%, with mixed pathogens or gram-negative organisms accounting for the majority of cases. There was no difference in adequate antibiotic treatment (refer to Table 2). Baseline laboratory data were comparable between the two groups and shown in Table 3. Patients in the Tα1 group had a lower level of mHLA-DR (47.1 vs. 58.0% in the control group, *P *= 0.02), but the distribution of each stratum in the two groups was similar.

**Table 1:** Baseline characteristics in both study groups.

	Control group	Tα1 group	*P *value
n	180	181	
Age (yr)	66.4 ± 12.6	64.7 ± 14.5	0.46
Age group			0.79
< 50 yr	21 (11.7%)	24 (13.3%)	
50-60 yr	39 (21.7%)	45 (24.9%)	
61-70 yr	39 (21.7%)	39 (21.6%)	
71-yr	81 (45.0%)	73 (40.3%)	
Male	131 (72.8%)	141 (77.9%)	0.26
Female	49 (27.2%)	40 (22.1%)	
BMI	22.0 ± 3.0	22.2 ± 3.1	0.48
**Prior or preexisting conditions**			
Congestive heart failure	8 (4.4%)	5 (2.8%)	0.39
Hypertension	79 (43.9%)	80 (44.2%)	0.95
Coronary heart disease	19 (10.6%)	22 (12.2%)	0.63
Liver disease	10 (5.6%)	9 (5.0%)	0.80
COPD	28 (15.6%)	29 (16.0%)	0.90
Nervous system diseases	33 (18.3%)	32 (17.7%)	0.87
Diabetes	34 (18.9%)	40 (22.1%)	0.45
Recent trauma	8 (4.4%)	8 (4.4%)	0.99
Cancer	55 (30.6%)	60 (33.2%)	0.60
**Recent surgical history**			0.47
No history of surgery	103 (57.2%)	92 (50.8%)	
Elective surgery	41 (22.8%)	46 (25.4%)	
Emergency surgery	36 (20.0%)	43 (23.8%)	
**Other indicators of disease severity**			
Mechanical ventilation	143 (79.4%)	146 (80.7%)	0.77
Shock	74 (41.1%)	64(35.4%)	0.26
Use of any vasopressor or dobutamine	72 (40.0%)	71(39.2%)	0.88
Low-dose corticoid	18 (10.0%)	20 (11.1%)	0.75
Blood transfusion	54 (30.0%)	64 (35.4%)	0.28
**Baseline acute organ dysfunctions**			
Pulmonary	170 (94.4%)	172 (95.0%)	0.80
Renal	48 (26.7%)	53 (29.3%)	0.58
Cardiovascular	113 (62.8%)	124 (68.5%)	0.25
Hematologic	69 (38.3%)	67 (37.0%)	0.80
Hepatic	39 (21.7%)	27 (14.9%)	0.10
**Number of acute organ dysfunction**			0.97
1	32 (17.8%)	29 (16.0%)	
2	75 (41.7%)	77 (42.5%)	
3	45 (25.0%)	48 (26.5%)	
4	18 (10.0%)	19 (10.5%)	
5	10 (5.6%)	8 (4.4%)	
**APACHE II score**	21.6 ± 7.7	22.3 ± 6.7	0.35
**SOFA score**	7.7 ± 3.9	7.9 ± 3.6	0.65
Respiratory system	2.6 ± 1.0	2.7 ± 0.9	0.22
Coagulation	0.8 ± 1.1	1.0 ± 1.2	0.17
Cardiovascular system	1.4 ± 1.6	1.2 ± 1.5	0.40
Liver	0.6 ± 0.9	0.5 ± 0.8	0.38
Nervous system	1.3 ± 1.4	1.4 ± 1.4	0.69
Renal system	1.0 ± 1.3	1.0 ± 1.4	0.85
Time from first organ dysfunction to enrollment (hr) median(IQR)	28.0 (15.0-48.0)	42.0 (24.0-72.0)	0.003

APACHE II, Acute Physiology and Chronic Health Evaluation II; BMI, body mass index; COPD, chronic obstructive pulmonary disease; IQR, interquartile range; SOFA, Sequential Organ Failure Assessment; Tα1, thymosin alpha 1; yr, year.

**Table 2:** Sites, causes of infection and adequate antibiotic treatment in patients with severe sepsis.

	Control group (*n *= 180)	Tα1 group (*n *= 181)	*P *value
Sites of infection*			
Lung	133 (73.9%)	136 (75.1%)	0.79
Abdomen	48 (26.7%)	51 (28.2%)	0.75
Urinary tract	5 (2.8%)	2 (1.1%)	0.28
Positive blood culture	10 (5.6%)	11 (6.1%)	0.83
Other^†^	18 (10.0%)	16 (8.8%)	0.71
**Results of pathogens**			0.99
Pure gram-negative	47 (26.1%)	51 (28.2%)	
Pure gram-positive	15 (8.3%)	14 (7.7%)	
Pure fungus	22 (12.2%)	21 (11.6%)	
Mixed	57 (31.7%)	56 (30.9%)	
Culture negative	39 (21.7%)	39 (21.6%)	
**Types of organisms‡**			
**Gram-positive**			
Staphylococcus aureus	7 (3.9%)	9 (5.0%)	0.62
Other staphylococcus species	9 (5.0%)	12 (6.6%)	0.51
Enterococcus species	22 (12.2%)	23 (12.7%)	0.89
Other gram-positive	18 (10.0%)	14 (7.7%)	0.45
**Gram-negative**			
Klebsiella species	18 (10.0%)	22 (12.2%)	0.51
Escherichia coli	25 (13.9%)	23 (12.7%)	0.74
Pseudomonas species	32 (17.8%)	32 (17.7%)	0.98
Acinetobacter	8 (4.4%)	15 (8.3%)	0.14
Enterobacter species	4 (2.2%)	4 (2.2%)	1.00
Other gram-negative	14 (7.8%)	16 (8.8%)	0.71
**Fungus**			
Candida albicans	43 (23.9%)	38 (21.0%)	0.51
Other candida species	20 (11.1%)	15 (8.3%)	0.36
Mould	1 (0.6%)	4 (2.2%)	0.37
Other fungus	6 (3.3%)	6 (3.3%)	0.99
**Empirical antibiotic therapy**			0.903
Adequate	136 (75.6%)	133 (73.5%)	
Inadequate	34 (18.9%)	37 (20.4%)	
Not evaluable	10 (5.6%)	11 (6.1%)	

*Patients may have had more than one site of infection; †other sites of infection included skin, central nervous system, bones and joints; ‡patients may have had more than one organism cultured. Tα1, thymosin alpha 1.

**Table 3:** Baseline levels of laboratory values.

	Control group	Tα1 group	*P *value
mHLA-DR (%)			
Median (IQR)	58.0 (33.9-83.0)	47.1 (26.4-71.1)	0.02
mHLA-DR group			0.16
< 30% (n, %)	36 (20.3%)	50 (27.6%)	
≥ 30- < 45% (n, %)	29 (16.4%)	32 (17.7%)	
≥ 45- < 85% (n, %)	70 (39.6%)	71 (39.2%)	
≥ 85% (n, %)	42 (23.7%)	28 (15.5%)	
CD^4+^/CD^8+ ^			
Median (IQR)	1.95 (1.18-3.30)	1.87 (1.16-3.22)	0.64
WBC (*10^9^)			
Median level	14.3 (10.1-17.9)	14.4 (9.4-19.3)	0.78
Neutrophil (%WBC)			
Median (IQR)	85.1 (80.2-90.7)	86.5 (80.8-91.0)	0.48
Lymphocyte (%WBC)			
Median (IQR)	9.5 (6.0-15.3)	8.9 (5.0-14.1)	0.23
Monocyte (%)			
Median (IQR)	4.80 (3.30-7.30)	4.95 (2.80-7.30)	0.66
Lactate (mmol/L)			
Median (IQR)	2.1 (1.4-3.4)	2.1 (1.3-3.1)	0.86

CD, cluster of differentiation; IQR, interquartile range; mHLA-DR, monocyte human leukocyte antigen-DR; WBC, white blood cell; Tα1, thymosin alpha 1.

### Study outcomes

#### Primary outcome

**Figure 2:**
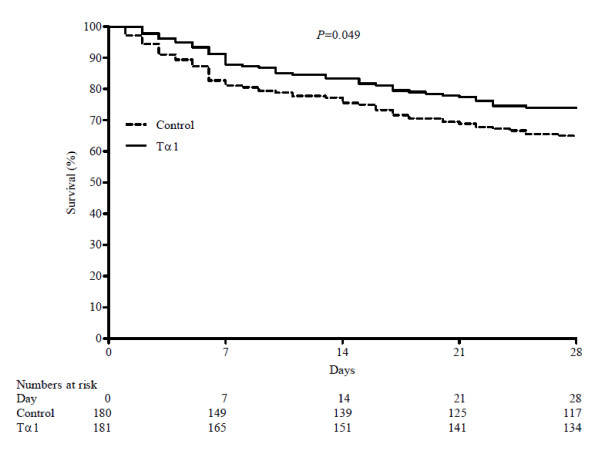
**Kaplan-Meier estimate of the probability of 28-day survival**. Tα1, thymosin alpha 1.

Within 28 days after the enrollment, 47 of 181 patients in the Tα1 group (26.0%) and 63 of 180 patients in the control group (35.0%) expired. The relative risk of death in the Tα1 group as compared to the control group was 0.74 (95% CI 0.54 to 1.02) with a *P *value of 0.062 in the nonstratified analysis. There was a 9.0% (95% CI -0.5 to 18.5%) absolute reduction in mortality in the Tα1 group. Survival time-to-event curves of the two groups are presented in Figure 2. Patients in the Tα1 group survived longer after enrollment than the control group (log rank, *P *= 0.049). A total of 52 of 181 patients in the Tα1 group (28.7%) and 71 of 180 patients in the control group (39.4%) died in hospital. The relative risk of death in hospital in the Tα1 group was 0.73 (95% CI 0.54 to 0.98) compared to the control group with a *P *value of 0.032. There was no significant difference in ICU mortality, ventilation-free days, ICU-free days, the length of ICU stay and duration of mechanical ventilation between the two groups (Table 4).

**Table 4:** Primary outcome and prognosis.

	Control group (*n *= 180)	Tα1 (*n *= 181)	*P *value
28-day mortality	63 (35.0%)	47 (26.0%)	0.062
In-hospital mortality	71 (39.4%)	52 (28.7%)	0.032
In-ICU mortality	48 (26.7%)	35 (19.3%)	0.098
Duration of ventilation			
Median (IQR)	6.0 (2.0-14.0)	7.0 (3.0-13.0)	0.742
ICU stay			
Median (IQR)	10.5 (5.0-20.5)	11.0 (7.0-20.0)	0.254
Ventilation-free days*			
Median (95% CI)	13.0 (7.0-18.0)	18.0 (15.0-21.0)	0.077
ICU-free days*			
Median (95% CI)	5.0 (0.3-10.7)	10.0 (6.8-15.0)	0.235

'Free days' were calculated as the number of days that the patient was alive and free of given measure (ventilator use and ICU stay) during the 28-day study period. CI, confidence interval; IQR, interquartile range; Tα1, thymosin alpha 1.

#### Secondary outcomes

Dynamic changes in SOFA and laboratory measurements are summarized in Table 5. A sustained increase in mHLA-DR values (% of positive monocytes) was observed in both groups. The mean changes from baseline on day 3 and day 7 were 4.1% and 11.2% in the control group, and 8.0% and 17.0% in the Tα1 group. Patients in the Tα1 group had lower baseline mHLA-DR than those in the control group on day 0. The average mHLA-DR became comparable with no statistically significant difference between the two groups on day 3 and 7. Greater improvements in mHLA-DR were observed in patients in the Tα1 group on day 3 (mean difference in mHLA-DR changes between the two groups was 3.9%, 95% CI 0.2 to 7.6%, *P *= 0.037) and day 7 (mean difference in mHLA-DR changes between two groups was 5.8%, 95% CI 1.0 to 10.5%, *P *= 0.017). The average SOFA score changes on day 3 and day 7 were -1.3 (95% CI -1.7 to -0.8, *P *< 0.001) and -1.8 (95% CI -2.4 to -1.3, *P *< 0.001) in the control group, and -1.8 (95% CI -2.3 to -1.4, *P *< 0.001) and -2.5 (95% CI -3.1 to -2.0, *P *< 0.001) in the Tα1 group. The decreasing tendency within 7 days in SOFA score seemed to favor the Tα1 group but with no significant difference in changes between the two groups. The ratio of CD^4+^/CD^8+ ^remained unchanged during the 7 days in both groups.

**Table 5:** Dynamic changes of SOFA and laboratory measurements.

Measures	Control group	Tα1 group	Between groups difference
	Mean (95% CI)	Mean (95% CI)	
SOFA score			
Day 0	7.7 (6.8-8.5)	7.9 (7.0-8.7)	
Day 3	6.4 (5.6-7.2)	6.1 (5.2-6.9)	
Day 7	5.9 (5.0-6.7)	5.3 (4.5-6.2)	
ΔDay 3*	-1.3 (-1.7--0.8)^a^	-1.8 (-2.3--1.4)^a^	-0.5 (-1.2-0.1)
ΔDay 7*	-1.8 (-2.4--1.3)^a^	-2.5 (-3.1--2.0)^a^	-0.7 (-1.5-0)
mHLA-DR (%)			
Day 0	58.2 (38.8-77.6)	51.8 (32.5-71.2)	
Day 3	62.2 (42.8-81.6)	59.8 (40.4-79.2)	
Day 7	69.4 (50.0-88.8)	68.9 (49.5-88.2)	
ΔDay 3*	4.1 (1.4-6.7)^b^	8.0 (5.4-10.5)^b^	3.9 (0.2-7.6)^a^
ΔDay 7*	11.2 (7.8-14.7)^b^	17.0 (13.7-20.3)^b^	5.8 (1.0-10.5)^a^
CD^4+^/CD^8+^			
Day 0	2.4 (2.0-2.9)	2.5 (2.0-2.9)	
Day 3	2.7 (2.2-3.1)	2.7 (2.3-3.2)	
Day 7	2.4 (2.0-2.9)	2.5 (2.1-3.0)	
ΔDay 3*	0.2 (0-0.5)	0.3 (0-0.5)^a^	0 (-0.3-0.4)
ΔDay 7*	0 (-0.3-0.3)	0.1 (-0.2-0.4)	0.1 (-0.3-0.5)

* ΔDay 3 and ΔDay 7 were defined as the value changes on day 3 and day 7 compared with that on day 0. ^a^*P *< 0.05; ^b^*P *< 0.01. CD, cluster of differentiation; CI, confidence interval; mHLA-DR, monocyte human leukocyte antigen-DR; SOFA, Sequential Organ Failure Assessment; Tα1, thymosin alpha 1.

#### Subgroup analysis

**Figure 3:**
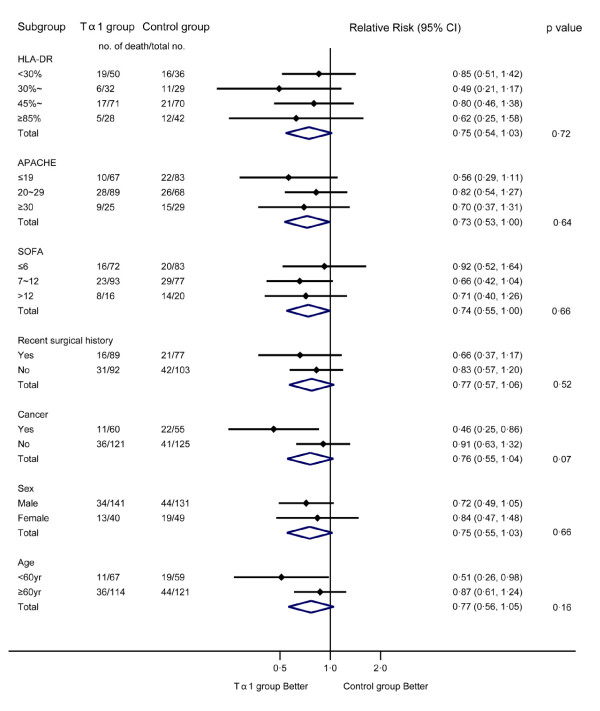
**Analysis of the rates and risks of death from any cause within 28 days in prespecified subgroups**. APACHE, Acute Physiology and Chronic Health Evaluation; CI, confidence interval; HLA-DR, human leukocyte antigen-DR; SOFA, Sequential Organ Failure Assessment; Tα1, thymosin alpha 1.

Mortality rates among prespecified subgroups of patients are shown in Figure 3. Prespecified analyses of the primary end point, where patients were stratified according to APACHE II score, SOFA score, mHLA-DR level, history of surgery or cancer, sex and age, showed that Tα1 tended to improve outcome but without statistical significance. In a subgroup analysis of patients with cancer, the relative risk of death of the Tα1 group when compared to the control group was 0.46 (95% CI 0.25 to 0.86, *P *= 0.01); on the other hand, in non-cancerous patients, the relative risk of death of the Tα1 group was 0.91 (*P *= 0.07 by the test of interaction).

#### Adverse events

Safety and tolerability assessment of Tα1 (see Additional file 3) was based on the comparison of all available information obtained from the two groups with respect to detected outliers in laboratory safety data, drug-related serious adverse events (assessed by the investigator) and deterioration of organ and system function (assessed by the individual SOFA component scoring for respiratory, cardiovascular, hepatic, coagulation, renal and nervous systems that arose during the treatment).

In this study, no Tα1-related severe adverse event (SAE) was reported and no treatment was discontinued due to intolerance or adverse events. There were no statistically significant differences between the control and Tα1 group with regard to the frequency of outlying laboratory values and all-cause organ or system impairment (refer to Table 6).

**Table 6:** Frequency of patients with outlying values of laboratory safety assays and all-cause organ and system impairment.

	Control group No. (%) (*n *= 180)	Tα1 group No. (%) (*n *= 181)	*P *value
**Laboratory safety assays**			
ALT (U/L)	43 (23.9)	38 (21.0)	0.51
AST (U/L)	44 (24.4)	43 (23.8)	0.88
Hypoglycemia	9 (5.0)	8 (4.4)	0.79
Hemoglobin (g/L)	23 (12.8)	27 (14.9)	0.56
Platelets (10^3^/mm^3^)	77 (42.8)	67 (37.0)	0.26
Creatinine (mmol/L)	12 (6.7)	18 (9.9)	0.26
SOFA component scores*			
Respiratory system	27 (15.0)	24 (13.3)	0.64
Coagulation system	52 (28.9)	48 (26.5)	0.62
Cardiovascular system	21 (11.7)	28 (15.5)	0.29
Hepatic system	25 (13.9)	21 (11.6)	0.51
Nervous system	22 (12.2)	14 (7.7)	0.15
Renal system	19 (10.6)	26 (14.4)	0.27

*Organ and system impairment based on the deterioration of SOFA component scores during the treatment. ALT, alanine aminotransferase; AST, aspartate aminotransferase; SOFA, Sequential Organ Failure Assessment; Tα1, thymosin alpha 1.

## Discussion

Immune system dysregulation plays a significant role in the course of sepsis. Previously, it was believed that the exaggerated pro-inflammatory response and its associated inflammation-induced organ injury were the major factors leading to deaths in sepsis. However, recent studies indicate that heterogeneity exists in septic patients' immune response, with some appearing immunostimulated, whereas in others appearing suppressed [23]. Although both pro-inflammatory and anti-inflammatory drugs have been evaluated, few have yet been found to significantly reduce the mortality [24-26]. Tα1 is thought to have immunomodulating effects primarily affecting the augmentation of T-cell function [27,28]. Tα1 has also shown actions beyond its effect on T lymphocytes by acting as an endogenous regulator of both the innate and adaptive immune systems [11,29]. Tα1 plays a unique role in balancing pro- and anti-inflammatory cytokine production through the involvement of distinct Toll-like receptors (TLRs) acting on different dendritic cells (DC) subsets and involving the MyD88-dependent signaling pathway. Tα1 can increase IL-12, IL-2, IFN-α and IFN-γ secretion to present antimicrobial effect and increase IL-10 and percentage of regulatory T cells (Tregs) to control inflammation [11,30-32]. Therefore, theoretically, Tα1 may be an appropriate immunoregulator for treating severe sepsis that is characterized by the large heterogeneity in immune function.

Our data suggested that the administration of Tα1 reduced 28-day mortality from any cause in patients with clinically diagnosed severe sepsis by 9.0%, with a marginal *P *value (*P *= 0.062 in the nonstratified analysis; log rank, *P *= 0.049) and decreased in-hospital mortality (*P *= 0.032). Our study was prospectively set up to detect an absolute 15% mortality reduction from an expected 50% as indicated in our previous trial and another epidemiology research about severe sepsis in China [22,33]. Mortality and drug effect size were not consistent with our expectation, which might lead to the marginal *P *value in the comparison of 28-day survival rate between two groups. In contrast to our results, previous trials in adults indicated that Tα1 significantly reduced mortality by 13.1% to 18% as compared to the control group [14-16]. The following reasons may explain this discrepancy in different trials. First, heterogeneity in patient populations and different therapeutic approaches could have influenced the outcomes; second, previous trials did not report the allocation concealment, which could have an unexpected impact on results. Schulz *et al. *indicated that the odds ratios were exaggerated by 41% for inadequately concealed trials and by 30% for unclearly concealed trials [34]. Third, those studies used more than one drug as therapeutic intervention and made it difficult to attribute the beneficial effects observed to each agent.

The most frequently assessed biomarker for evaluating immune function of severe sepsis is mHLA-DR. There seems to be a general consensus that diminished mHLA-DR is a reliable marker for the development of immunodysfuction in severe sepsis patients [35,36]. Recent studies indicate that the dynamic change of mHLA-DR over time was a better predictor of mortality and mHLA-DR recovery was associated with a better prognosis [21,37,38]. In the present trial, a greater improvement of mHLA-DR was observed in the Tα1 group on day 3 and day 7 than in the control group, which suggests that Tα1 may improve immune function in severe sepsis. The ratio of CD^4+^/CD^8+ ^is another parameter to evaluate immunological status in sepsis. Decreased CD4^+^/CD8^+ ^ratio was related to the development of severe sepsis and multiple organ failure (MOF) in trauma patients [39]. Some studies showed that thymosin alpha 1 can increase CD4^+^/CD8^+ ^ratio [40,41]. On the other side, one research study has indicated that mHLA-DR, not CD^4+^, CD^8+ ^or ratio of CD^4+^/CD^8+^, can predict the prognosis of severe sepsis [42]. In our research, we did not find statistically significant difference in the CD4^+^/CD8^+ ^ratio between the two groups. The decreasing tendency within 7 days in SOFA score seemed to favor the Tα1 group but with no significant difference in changes between the two groups. However, considering the fact that we observed the changes of these indices for only 7 days, there could have been some difference between the two groups if the observation had been extended to 14 or 28 days.

The median time from the first organ dysfunction detected to enrollment was more than 24 hrs in both groups, but longer in the Tα1 group. We adopted a retrospective method to determine the time window between the onset of the first organ dysfunction detected and study enrollment according to objective data (such as blood gas analysis), many of which were obtained before transferring the severe sepsis patients to the ICU [7]. However, those patients without indicative objective data could also have suffered from severe sepsis and the delay in laboratory tests could substantially underestimate the time after onset. In other words, the time after onset determined by laboratory tests in non-ICU departments was out of our control and subject to errors, especially when the estimation was based on hours instead of days. The precise time window between onset of the first organ dysfunction and enrollment could exceed the recorded time and could possibly be balanced between the two groups. The better way of enrolling severe sepsis patients in immunotherapy research may be through mHLA-DR value, which has been proved to be a good predictor to evaluate patients' immune status and a good parameter for individualized goal-directed therapy [43].

Reductions in the relative risk of death were observed in all subgroups including those stratified according to age, sex, APACHE II score, SOFA score and levels of mHLA-DR, but without statistical significance. The aim of analyzing different prespecified subgroups in our research was to prepare for our future research in targeted specific groups of severe sepsis patients who might benefit from the Tα1 treatment since it is unlikely that thymosin alpha 1 is equally beneficial to all patients in view of the significant heterogeneity in severe sepsis patients. The results of subgroup analysis in our research were inconclusive and whether Tα1 is more effective in specific groups of patients with severe sepsis should be explored in trials with a larger sample size.

Types of pathogen and empirical antibiotic therapy are very important factors that affect the outcome of severe sepsis. In our study, there was no difference between groups in these perspectives. It is noted that the origins of microorganisms are substantially diverse in different areas and even in different hospitals in the same area. So is empirical therapy. In the present study, there was a high isolation rate of gram-negative bacteria (pseudomonas, acinetobacter) compared with some other epidemiology study of infection in ICU [44]. In fact, the relatively higher incidence of pseudomonas and acinetobacter infections is not unusual in China [33] so that the adequate empirical therapy is adjusted accordingly.

Thymosin alpha 1 has been shown to be a safe and well-tolerated agent in other studies [12,13]. Serious adverse events were not observed in our trial. Outlying laboratory values and all-cause organ and system impairment were similar in both groups. However, subjective sensations such as irritation or burning, general or gastrointestinal disorders were difficult to assess due to the severity of disease, sedation or analgesia in severe sepsis patients.

In our study, several factors limit the extent to which the results can be generalized. First, the study population was heterogeneous with respect to clinical features. Although over 80 baseline characteristics were comparable between the two groups, difference in mHLA-DR expression was present and was probably due to the heterogeneity in patients and the relatively small size of samples. In fact, unbalanced baseline characters between groups were not rare in severe sepsis trials even with large samples [45,46]. In our study, to assess whether outcomes differed by treatment groups, linear mixed models for longitudinal data were fit with adjustment for the baseline value. This method has been widely used in multicenter research [47,48]. Second, considering the heterogeneity of severe sepsis, some patient groups could benefit more from the intervention than other septic patients. The future individualized and goal-directed Tα1 treatment of severe sepsis should be implemented in targeted specific groups of patients. One of the biomarkers that can be used to stratify patients according to their immune status is mHLA-DR. Meisel *et al. *reported that mHLA-DR level was associated with immunosuppression status in sepsis patients who benefited from the granulocyte-macrophage colony-stimulating factor (GM-CSF) treatment [43]. We will try to adopt mHLA-DR target immunosuppression patients in future study. Third, since a considerable proportion of patients were transferred out of ICU within one week, which makes it difficult to guarantee that the complete laboratory and follow-up data could be obtained, we only collected laboratory data within 7 days and followed up the survival status for 28 days. A more extensive laboratory data collection and extended follow-up period could possibly provide more significant information. Fourth, there are few biomarkers to evaluate the immunological derangement. In the present trial, we adopted the widely used mHLA-DR. Fifth, it is not known from our trial that whether the extension of the treatment to more than 7 days or the increase of dose could generate a significant improvement in the outcomes of severe sepsis patients. Sixth, we did not adopt the double-blind method because no identical-appearing placebo was available and only the patients and the statistician were blinded. To minimize the potential bias, randomization and adequate allocation concealment were meticulous in the trial [34] and the primary and second end points were objective rather than subjective.

Given these limitations, the present research is a preliminary exploration on the efficacy of thymosin alpha 1 in severe sepsis and further double-blinded studies are needed to explore the use of Tα1 regarding patient selection, dosage and the course of treatment.

## Conclusions

This RCT demonstrates that thymosin alpha 1 therapy in combination with conventional medical therapy may be effective in improving clinical outcomes in a targeted population of severe sepsis. Larger multicenter studies are indicated to confirm these findings.

## Key messages

• In light of the crucial role of immunologic derangement in severe sepsis, immunotherapy may be an important adjunctive treatment.

• This study demonstrates that immunodulation with thymosin alpha 1 may effectively improve outcomes of patients with severe sepsis. A beneficial impact on the immunofunction of patients with severe sepsis was also observed. Further researches are needed to confirm these findings.

## Abbreviations

ALT: alanine aminotransferase; APACHE II: Acute Physiology and Chronic Health Evaluation II; AST: aspartate aminotransferase; BMI: body mass index; CD: cluster of differentiation; CI: confidence interval; ICU: Intensive Care Unit; IFN: interferon; IL: interleukin; IQR: interquartile range; *P*: *P *value; mHLA-DR: monocyte human leukocyte antigen-DR; OR: odds ratio; SIRS: systemic inflammatory response syndrome; SOFA: Sequential Organ Failure Assessment; Tα1: thymosin alpha 1; WBC: white blood cell.

## Competing interests

The authors declare that they have no competing interests.

## Authors' contributions

JW, XG designed the research; JC, BO, MC, LZ, YL, XQ, JL, XW, YC, GM, BG, QK, LC, ZH, ZZ performed the research and collected data; AL, GZ analyzed the data; JW wrote the manuscript. All authors read and approved the final manuscript.

## Supplementary Material

Additional file 1 **Study inclusion criteria**. The detailed criteria to be fulfilled for study inclusion. Click here for file

Additional file 2 **Study exclusion criteria**. Patients who met the criteria were excluded. Click here for file

Additional file 3 **Safety and tolerability assessment of thymosin alpha 1**. Safety and tolerability assessment of thymosin alpha 1 was based on the comparison of all available information obtained from the two groups with respect to detected outliers in laboratory safety data, drug-related serious adverse events and deterioration of organ and system function. Click here for file
