# Supplementary material for: The efficacy of thymosin alpha 1 for severe sepsis (ETASS): a multicenter, single-blind, randomized and controlled trial
Source: Crit Care. 2013 Jan 17;17(1):R8. doi: 10.1186/cc11932 (PMC4056079; doi:10.1186/cc11932)
Supplement: Additional file 1 — Study inclusion criteria. The detailed criteria to be fulfilled for study inclusion. [file cc11932-S1.DOCX]

Additional file 1. Study inclusion criteria

The following criteria had to be fulfilled for study inclusion (1-3):

1. Written informed consent from the patients or their next of kin for patients unable to consent

2. Age ≥18 yrs and ≤ 85 yrs

3. Presence of severe sepsis/ septic shock (A-C required):

*A)* evidence of infection: patients have to have a known infection or a suspected infection, as evidenced by one or more of the following: white cells in a normally sterile body fluid; perforated viscus; radiographic evidence of pneumonia in association with the production of purulent sputum; a syndrome associated with a high risk of infection (e.g., ascending cholangitis).

*B)* two or more of the following criteria of SIRS criteria are met: fever (> 38°C), hypothermia (< 36°C); tachycardia (> 90 beats/min), except in patients with a medical condition known to increase the heart rate or those receiving treatment that would prevent

tachycardia; tachypnea (> 20 breaths/min), or PaCO_2_ < 32 mmHg, or the use of mechanical ventilation; leukocytosis (> 12,000/ mm^3^), leukopenia (< 4,000/ mm^3^), or > 10 % immature neutrophils.

*C)* acute organ dysfunction (at least one):

- for cardiovascular system dysfunction: the arterial systolic blood pressure had to be < 90 mmHg or decrease in systolic arterial pressure > 40 mmHg or the mean arterial pressure < 65 mmHg for at least 1 hour despite adequate fluid resuscitation, adequate intravascular volume status or the use of vasopressors in an attempt to maintain a systolic blood pressure of ≥ 90 mm Hg or a mean arterial pressure of ≥ 65 mm Hg;

- for kidney dysfunction: urinary output < 0.5ml/kg/h for > 2 h or creatinine increase > 2mg/dl in 48 hrs, or need for renal replacement therapy

- for respiratory system dysfunction: the ratio of PaO_2_ to FiO_2_ had to be ≤ 300;

- for hematologic dysfunction: platelet count less than 100,000/mm^3^ or INR > 1.5 or aPTT > 60 s;

-for hepatic-system dysfunction: total bilirubin >2.0 mg/dl or 35 mmol/l;

-metabolism: pH had to be ≤ 7.30 or the base deficit had to be ≥5.0 mmol/liter in association with a plasma lactate >3 mmol/l.
