# Supplementary material for: The efficacy of thymosin alpha 1 for severe sepsis (ETASS): a multicenter, single-blind, randomized and controlled trial
Source: Crit Care. 2013 Jan 17;17(1):R8. doi: 10.1186/cc11932 (PMC4056079; doi:10.1186/cc11932)
Supplement: Additional file 2 — Study exclusion criteria. Patients who met the criteria were excluded. [file cc11932-S2.DOCX]

Additional file 2. Study exclusion criteria

1. Pregnant or lactation period.
2. Age <18 yrs or >85 yrs
3. Receiving immunosuppressive therapy such as cyclosporine, azathioprine or cancer chemotherapy within one month.
4. History of bone marrow, lung, liver, kidney, pancreas or small bowel transplantation;
5. Acute pancreatitis with no established source of infection.
6. Not expected to survive 28 days because of end-stage diseases.
7. Participation in another clinical trial.
