# Supplementary material for: The efficacy of thymosin alpha 1 for severe sepsis (ETASS): a multicenter, single-blind, randomized and controlled trial
Source: Crit Care. 2013 Jan 17;17(1):R8. doi: 10.1186/cc11932 (PMC4056079; doi:10.1186/cc11932)
Supplement: Additional file 3 — Safety and tolerability assessment of thymosin alpha 1. Safety and tolerability assessment of thymosin alpha 1 was based on the comparison of all available information obtained from the two groups with respect to detected outliers in laboratory safety data, drug-related serious adverse events and deterioration of organ and system function. [file cc11932-S3.DOCX]

1. Additional file 3. Safety and tolerability assessment of Tα1

-For hematology assays, outliers were patients whose hemoglobin or platelet values fell to <75% of the lower limit of the normal range.

-For serum chemistry assays, outliers were those patients whose alanine aminotransferase (ALT), aspartate aminotransferase (AST), creatinine (Cr) values were normal at the baseline and rose to more than two times the upper limit of the normal range. For patients with abnormal baseline values, outliers were those whose assay values rose to more than one and a half times their baseline values.

-Any worsening SOFA scores identified were compared between the two groups to evaluate whether there was a difference in organ and system impairment.

-Severe adverse events were defined as medical occurrence that: a) led to a death; or b) led to a serious deterioration in health that resulted in one or more of the followings: 1) a life-threatening illness or injury, 2) a permanent impairment of a body structure or a body function, 3) in-patient hospitalization for treating related adverse events or prolongation of existing hospitalization, 4) medical or surgical intervention to prevent life threatening illness or injury or permanent impairment to a body structure or a body function.
